# The fate of germ cells in cryptorchid testis

**DOI:** 10.3389/fendo.2023.1305428

**Published:** 2024-01-03

**Authors:** Jorgen Thorup, Simone Hildorf, Andrea E. Hildorf, Jonas M. Baastrup, Linn Salto Mamsen, Claus Yding Andersen, Tina E. Olsen, Dina Cortes

**Affiliations:** ^1^Department of Pediatric Surgery, Surgical Clinic C, Copenhagen University Hospital Rigshospitalet, Copenhagen, Denmark; ^2^Department of Clinical Medicine, University of Copenhagen, Copenhagen, Denmark; ^3^The Laboratory of Reproductive Biology, Rigshospitalet, Copenhagen, Denmark; ^4^Department of Pathology, Copenhagen University Hospital, Rigshospitalet, Copenhagen, Denmark; ^5^Department of Pediatrics and Adolescent Medicine, Copenhagen University Hospital Hvidovre, Hvidovre, Denmark

**Keywords:** germ cell, male infertility, testicular cancer, cryptorchidism, testes (Source: MeSH)

## Abstract

Cryptorchidism in males constitutes a notable risk factor for both infertility and testicular cancer. Infertility in adulthood is closely linked to the germ cell status in childhood. Furthermore, the significance of germ cell status is important as more than 95% of all reported testicular malignancies are germ cell tumors. The review aims to elucidate the pathogenesis of germ cells in cryptorchid testes concerning their association with infertility and testicular malignancies. Impaired germ cell numbers are evident in cryptorchid testes even during antenatal and neonatal stages. In cryptorchidism there is a rapid decline in germ cell number within the first year of life, partially attributed to physiologic gonocyte apoptosis. Additionally, germ cells fail to differentiate normally during mini-puberty leading to reduced germ cell proliferation and delayed clearance of gonocytes from the seminiferous epithelium. Absence of germ cells in testicular biopsies occurs already 10 months of age and germ cell deterioration progressively worsens with approximately 50% of persisting cryptorchid testes lacking germ cells during puberty. The deficient germ cell maturation and proliferation leads to later infertility. Elevated temperature in the cryptorchid testes and also hormonal deficiency contribute to this phenomenon. Germ cell neoplasia *in situ* (GCNIS) originating during fetal development may manifest in rare cases associated with disorders of sexual development, chromosomal abnormalities in boys, specific syndromes, and teratomas that include cryptorchidism. In adults, the presence of GCNIS predominantly represents a new histology pattern before invasive germ cell cancer is demonstrated and is neither congenital nor related to abnormal gonocyte transformation.

## Introduction

Cryptorchidism is the most prevalent congenital anomaly in boys and represents a well-characterized risk factor for infertility and testis cancer. Men with a history of cryptorchidism constitute a substantial proportion of 20%–27% of azoospermic men (1–3). Furthermore, men with a prior history of bilateral cryptorchidism exhibit considerably lower paternity rates, at 65% compared to men who experienced unilateral cryptorchidism (90%) and unaffected control men (93%) (3–5). The evidence is compelling that infertility in adulthood has a clear relation to the germ cell status in childhood (3, 6–15). In addition, boys with cryptorchidism face an elevated risk of testicular neoplasia later in life surpassing the risk observed in the general population (3, 16–21). Notably, the status of the germ cells is important, given that germ cell tumors constitute more than 95% of all reported testicular malignancies (22, 23). In recognition of their connection with germ cell neoplasia *in situ* (GCNIS), which can be identified in cryptorchid testes, the World Health Organization (WHO) classified testicular germ cell tumors into GCNIS derived tumors (seminomas, non-seminomas) and non-GCNIS derived tumors (spermatocytic tumors, prepubertal-type germ cell tumors) (24).

The primary objective of this review is to elucidate the histological pathology of germ cells in cryptorchid testes in comparison to the normative course of germ cell development. Moreover, we aim to delineate the germ cell pathogenesis in cryptorchid testes within the context of infertility and the development of testicular malignancies.

## Normal germ cell development

For obvious reasons there are limited normal reference materials available regarding human testicular germ cell development.

The sex-specific development of the male germline initiates around 6–7 weeks post-conception. At this point, primordial germ cells are settled in the gonadal ridge, and are now commonly referred to as gonocytes. The gonocytes begin to differentiate, proliferate and populate the seminiferous cords along with Sertoli cells (3, 25). Other classifications of gonocytes and primordial fetal germ cells, for example, pro- or prespermatogonia have been proposed (3, 26, 27), but no ubiquitous consensus regarding terminology has been reached. During the first trimester, gonocytes are mitotically active forming a quite homogenous cell population expressing markers typical of pluripotent cells including primordial germ cells such as KIT proto-oncogene (c-KIT), octamer-binding transcription factor 3/4 (Oct3/4), lin-28 homolog A (LIN28), homeobox protein Nanog (NANOG), anti-podoplanin M2A antigen (D2-40), and placental alkaline phosphatase (PLAP) (3, 25, 28–33). This suggests that gonocytes are quite equivalent to primordial germ cells, both presenting the distinctive morphology of being large spherical cells with a prominent nucleus containing one or two nucleoli surrounded by a spherical shape cytosol (3, 26, 34). Gonocytes occupy the center of seminiferous cords, that have no lumen yet.

From 5 to 19 weeks post-conception the number of germ cells increase from a mean of 3.700 to 1.417.000 based on stereological estimations of fetal human testes (35). As pregnancy nears its end, most fetal germ cells cease mitotic activity. Currently, they lose pluripotency and fetal markers leading to the formation of distinct germ cell subpopulations. They transform into fetal spermatogonia which are larger, flattened cells located on the basement membrane (36). Simultaneously, they begin to express additional germ cell-specific markers such as melanoma-associated antigen A4 (MAGE-A4), dead-box helicase 4 (DDX4; also known as the *Drosophilias* gene *vasa*, VASA), and deleted in azoospermia like (DAZL) (3, 29, 31). Importantly, in the testis from a fetus aged 18-weeks of gestation, VASA-positive germ cells were distributed more in the peripheral zones of the seminiferous tubules in contrast to the Oct3/4-positive germ cells. Collectively, these findings support the notion that male fetal germ cells undergo a progression of migration, mitosis, and cell-cycle arrest.

Postnatally, the germ cells continue to differentiate and migrate toward the basement membrane of the seminiferous tubule, forming the spermatogonia including the spermatogonial stem cell (SSC) population. This primarily takes place during the so-called minipuberty at 2-4 months of age. Three morphologically distinct types of spermatogonia have been classified, namely type A dark (Ad), type A pale, and type B spermatogonia (3, 37–39). It is believed that gonocyte transformation into Ad spermatogonia is an essential step for the formation of the SSC pool (3, 40, 41). Ad spermatogonia are believed to be representative of the SSCs. They can either self-renew to maintain the SSC pool or differentiate into A pale spermatogonia. The A pale spermatogonia can then undergo one or more divisions before differentiating into B spermatogonia (38). The nucleus of the Ad spermatogonia is homogeneous dense and dark featuring at least one rarefaction zone. This distinguishes it from the lighter stained, coarser nuclei from the other two types, which lack these rarefaction zones (3, 38, 39, 42). Ad spermatogonia usually appear during minipuberty, experiencing a marked increase in number by 3-4 months of age, after which they remaine predominantely quiescent (3, 43, 44), until puberty where they begin to differentiate, truly indicated by the ability to sustain meiotic division (25). Some fetal germ cells seem to persist post-birth and continue to be present up to the first months of life. This is evidenced by the presence of germ cells expressing the mainteinance markers D2-40, Oct3/4, cKit, and NANOG (3, 45–49). Kvist and co-workers (46, 47) found D2-40 positive germ cells up to 6 months of age, Oct3/4 up to 6-9 months of age, and c-Kit up to 11–16 years of age. Gonocytes that fail to migrate to the basement membrane and differentiate normally undergo apoptosis and are cleared from the seminiferous epithelium, which presumably takes place during minipuberty and up until 1 year of age (39, 50, 51). The importance of Ad spermatogonia has been supported by follow-up demonstrating their link with fertility outcomes (14, 15, 41). Ad spermatogonia have been found to be positive for PLAP and undifferentiated embryonic cell transcription factor 1 (UTF1) (3, 52, 53). However, though previously mentioned in the review of human spermatogonial markers by von Kopylow and co-workers (54), no specific marker for only Ad spermatogonia has been identified (3). Therefore, the identification of Ad spermatogonia relies on morphological criteria. Transformation of spermatogonia to primary spermatocytes usually begins around 3–4 years of age. Primary spermatocytes, which are in the first phase of meiotic division, exhibit a round nucleus with a chromatin pattern that reflects the stage of meiotic progression (36, 39, 40). During childhood, the germ cells express various different markers, such as c-KIT, UTF1, PLAP, MAGE-A4, VASA, and fibroblast growth factor receptor 3 (FGFR3). However, this is based on limited material since normal testis materials during the prepubertal period are sparse (3, 46, 49, 55).

**Figure 1:**
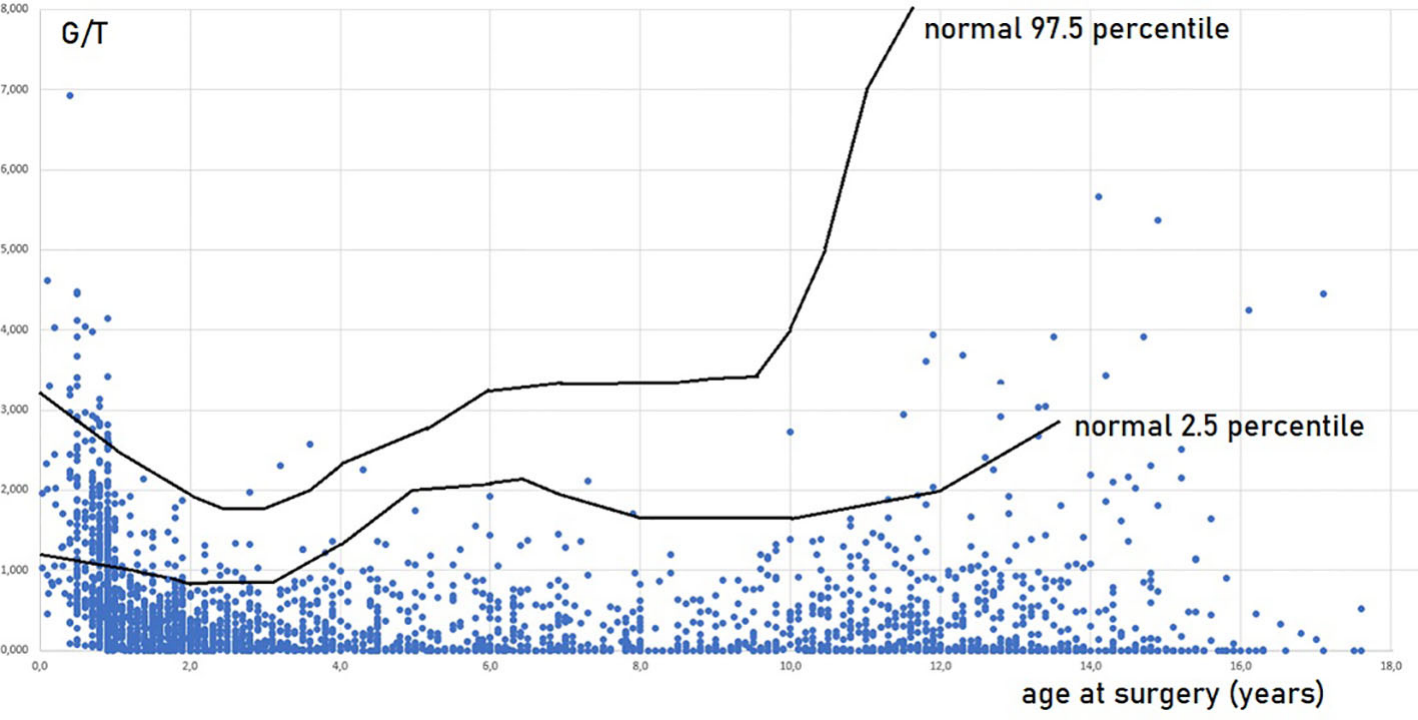
Germ cell number per tubular cross section (G/T) in testicular biopsies of 2410 boys operated for cryptorchidism in relation to age at surgery and normal values. Every boy appears with one value, in cases of bilateral cryptorchidism and bilateral biopsies a mean G/T value is shown. Most of the data are included in Cortes, 1998 (36) and Hildorf, 2022 (3). The normal values are in accordance with Masliukaite et al, 2016 (59).

The prepubertal testis has previously been regarded as a quiescent organ but serves as a crucial period for germ cell development. Proliferation and differentiation take place in the prepubertal testis as the number of germ cells varies a lot from mid-gestation toward puberty (56). From gestational week 28 until around 3 years of age, the total number of germ cells increase by a factor of 3 was demonstrated during the first 100 days of life with a maximum at 100–150 days of age and followed by a decrease by a factor of 0.5 based on stereological estimations of testes from boys who suffered from sudden death (51). The histological parameter, the number of germ cells per cross-sectional tubule (G/T), has been used in the quantification of germ cells and correlates with the number of germ cells per cm^3^ testicular parenchyma (56). Hence, G/T is a parameter of numerical density. Using normal materials, studies have shown that G/T levels quickly decline during the first 1-2 years of life. Subsequently, G/T numbers rise until they approximate birth levels at around 5- 6 years of age. These levels then continue to rise until 7 years, followed by a slight decline or plateau, and then a notable increase as puberty approache (3, 39, 56–58). The 95% confidence interval of normal G/T redrawn according to the meta-analysis by Masliukaite et al. (58) is seen in **Figure 1**. At the onset of puberty, the organization of the prepubertal testis changes dramatically as the Sertoli cells mature and form the blood–testis barrier by compartmentalizing the seminiferous epithelium into a basal and a luminal compartments, and the lumen occurs (36). These changes enable spermatogenesis.

## The fate of germ cells in cryptorchid testes in respect of infertility

**Figure 2:**
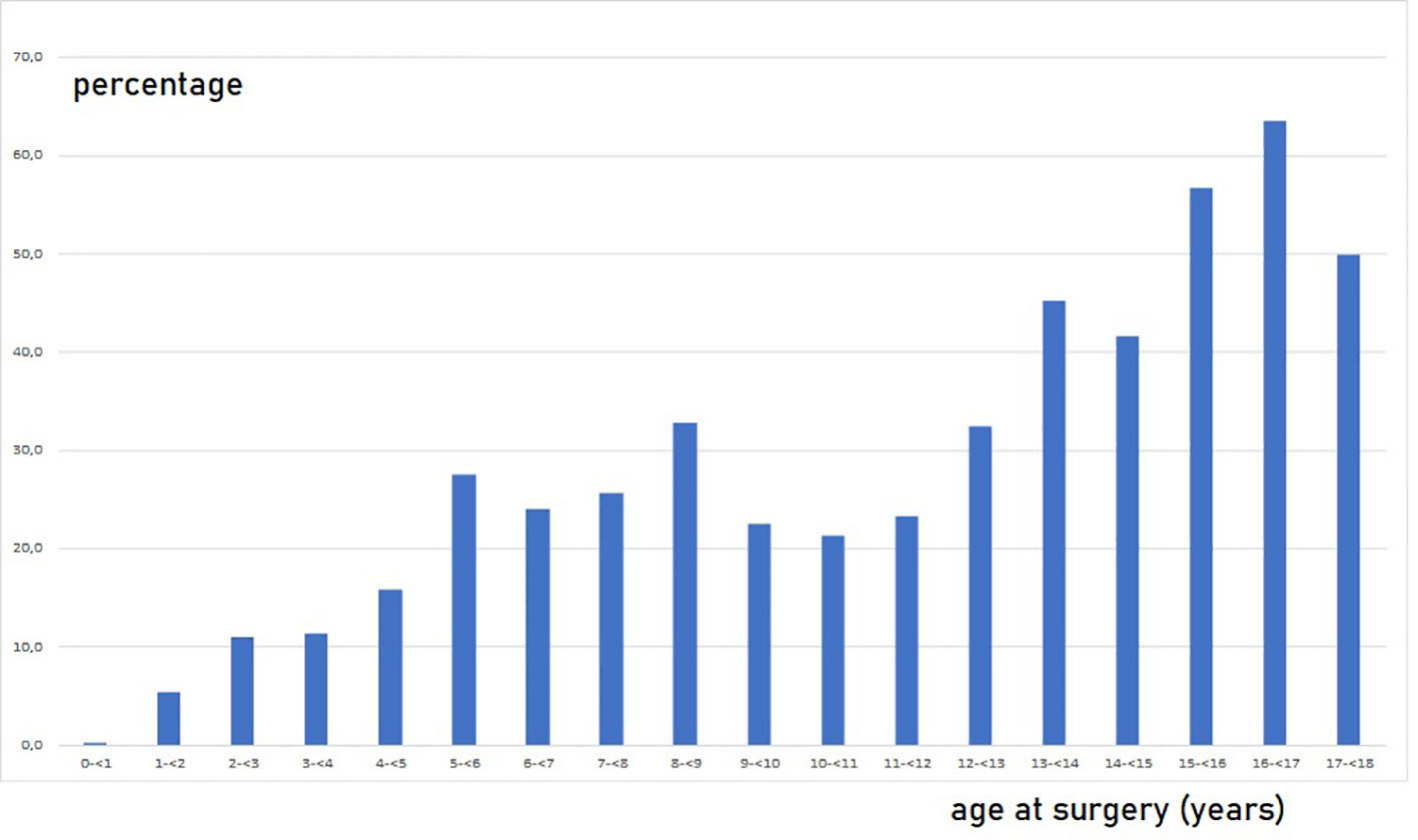
Percentage of boys without germ cells, Sertoli cell only, in testicular biopsies among 2410 boys operated for cryptorchidism in relation to age at surgery. Every boy appears with one value. In cases of bilateral cryptorchidism and bilateral biopsies, it is shown if one of the biopsies had Sertoli cells only. Most of the data are included in Cortes, 1998 (36) and Hildorf, 2022 (3).

**Figure 3:**
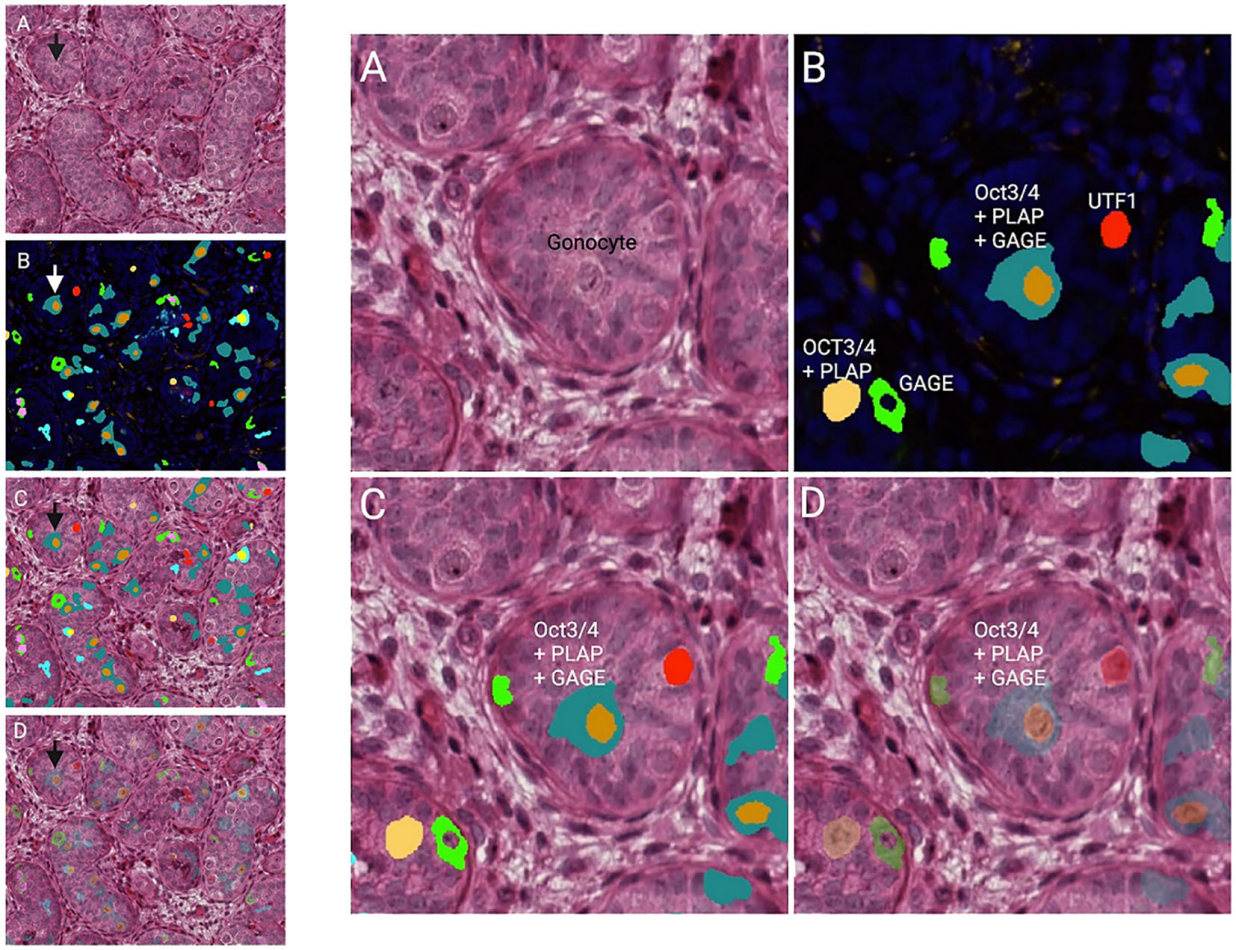
Testicular histology of a cryptorchid testis from a 9 months old boy. The arrows in the overview histology slides with lower magnification to the left point on the gonocyte in the center of the same histology slides with higher magnification to the right. **(A)** HE (hematoxylin-eosin). **(B)** DAPI (nucleui marker) and multiplex with immunoflourence (IF) markers against Oct ¾, PLAP, UTF, and GAGE. The specific color is either one of the markers or a profile of more. **(C, D)** HE overlap with IF markers (**D**: showing the HE morphology underneath the multiplex analysis).

Impaired G/T in cryptorchid testes can be congenital as it is identified already antenatally in the last trimester and in newborns (57) (**Figure 1**). In the study by Cortes et al. (57) of 35 third-trimester fetuses with cryptorchidism compared with 22 fetuses with normal descended testes, significantly reduced G/T values and lower testicular weights were found in the group of fetuses with cryptorchidism. All exhibited germ cells, but 23% had G/T below the lower normal range. Other anomalies, such as dysplasia of the kidneys, ureter or T10 to S5 vertebrae, were found in 34% of these fetuses with cryptorchidism. It is well documented that dysplastic changes of the kidney and ipsilateral cryptorchidism are related (59). Recent research indicates that impaired prenatal androgen action can be associated with unilateral cryptorchidism diagnosed during the first year of life (60). As evident from **Figure 1**, a substantial part of cryptorchid testes exhibit impaired G/T by the time the boy reaches one year of age. The youngest boy with Sertoli cell only in a testicular biopsy was 10 months old and had non-syndromic unilateral cryptorchidism with testis at the level of the external inguinal annulus (**Figure 2**). There is a fast decline in G/T during the first year, that to some extent is physiologic due to the apoptosis of the gonocytes that do not differentiate normally during mini-puberty, but fail to migrate to the basement membrane (3, 39, 50, 51) (**Figure 1**). There is also deficient germ cell proliferation and a delay in the process of clearing the gonocytes from the seminiferous epithelium. Oct3/4, LIN28 and D2-40 which are typical markers of pluripotent fetal germ cells and gonocytes stain some germ cells positive in cryptorchid testes of boys up to 20 months of age (**Figure 3**) (61–63). The percentage of germ cells with positive markers Oct3/4, LIN28 and D2-40 in cryptorchid testes of boys between the ages of 12 and 20 months was 13%, 19% and 3% respectively (61–63). Already between 2 and 3 years of age most boys with cryptorchidism have impaired G/T, and more than 10% experience depletion of germ cells showing Sertoli cell only, in testicular biopsies when orchidopexy is performed (**Figures 1**, **2**). The age-related progressive deterioration, both in terms of G/T and the percentage of testes with Sertoli cell only is illustrated in **Figures 1**, **2**. Bilateral Sertoli cell only leads to infertility (36). Whereas unilateral cryptorchidism with Sertoli cell only in the cryptorchid testis is associated with a 33% risk of later infertility (36). There are several different reasons for the germ cell deterioration in cryptorchid testes during childhood. An important issue is probably the testicular temperature. The normal testis descends from within the abdomen to the subcutaneous scrotum, enabling the testis to reside in a specialized, low temperature environment of approximately 33°C. Heat stress is suggested to be a major factor causing testicular germ cell abnormalities, given that the cryptorchid testis is not located in the scrotum, which maintains a cooler temperature (64). Temperatures exceeding those of the scrotum trigger cell death, and excessive death of germ cells has been proposed as being the cause of infertility seen in cryptorchid testes (64). It is not known when the descended human testis adapts to the scrotal environment and ambient temperature of 33°C, but it has been suggested that this normally occurs shortly after birth (65). COX-2, an inhibitor of apoptosis was elevated in cryptorchid testes, perhaps as a mechanism of preserving germ cell numbers [66].

Huff and co-workers (45) proposed that the abnormalities observed in cryptorchid testes were not owing solely due to a thermal injury based on analysis of nearly 800 human testicular biopsies from both the cryptorchid and contralateral descended testes. They showed that gonocytes failed to disappear and Ad spermatogonia failed to appear in cryptorchid testes below 1 year of age indicating a defect in the first maturation step at 2–3 months of age. This resulted in the failure to establish an adequate adult germ stem cell pool. Primary spermatocytes also failed to appear in cryptorchid testes and were found in only 19% of contralateral descended testes at 4–5 years of age suggesting a defect in the onset of meiosis. Moreover, the defects in the two prepubertal steps in germ cell maturation were associated with reduced total germ cell counts. Furthermore, it was proposed that also deficiency in the hypothalamic-pituitary axis may be responsible for these abnormalities.

Studies from our group support the finding by Huff and co-workers (45). Already at term, around 20% of fetuses with cryptorchid testes had germ cell hypoplasia, and G/T was below the lowest normal value for gestational age (57). Furthermore, a normal number of Ad spermatogonia was only found in 43% of cryptorchid testes from infant boys with normal germ cell numbers, but in none from testes of age-matched cryptorchid boys with germ cell hypoplasia (53). Hadziselimovic proposes that infertility in patients diagnosed with cryptorchid testes is the consequence of a hormonal deficiency rather than temperature-induced cellular damage (67). He argues that the transformation into Ad spermatogonia starts during mini-puberty and is a continuous gonadotropin dependent process during the prepubertal period. In a long-term study by Hadziselimovic and co-workers (14) they analyzed 89 cryptorchid boys having orchidopexy with a testicular biopsy taken between the ages of 1 to 16 years with follow-up in adult age. No hormonal treatment was performed prior to the surgery. In cases where Ad spermatogonia were depleted in both testes, none of the patients later exhibited a normal sperm count, even after successful surgery and despite the descended testes being subjected to cooler temperatures. Furthermore, in patients with bilateral cryptorchidism having Ad spermatogonia in one or both testes, 55% later developed a normal sperm count. In patients with the presence of Ad spermatogonia in both testes, 84% later had a normal sperm count, illustrating that the presence of Ad spermatogonia is an important predictor of male fertility (14). The importance of a post-pubertal gonadotropin deficiency became clear as inverse correlations were detected between FSH and sperm concentration, between FSH and Ad spermatogonia content of the cryptorchid testes, and positive correlations were found between FSH and LH levels, and between LH and presence of Ad spermatogonia. At least 70% of the patients without Ad spermatogonia at time of orchidopexy experienced a relative FSH deficiency in adulthood (14).

Our group’s findings partly corroborate the results of the previous study. We examined a series of 9 boys with bilateral cryptorchidism, suspected gonadotropin deficiency and no hormonal treatment. These boys underwent a re-biopsy performed at a median age of 32 months following orchidopexy accompanied by an initial testicular biopsy (**Table 1**) (68). In half of the patients there was no significant improvement of the germ cell status though the testes were satisfactorily positioned in the scrotum. In a follow-up study of 208 boys after bilateral orchidopexy the serum level of inhibin-B secreted by Sertoli cells was used as a marker of the status of the germinative epithelium. An overall improvement was demonstrated after positioning of the testes in the scrotum. The best results were seen if orchidopexy was performed in the first year of life. Furthermore, in boys with intact hypothalamic-pituitary-gonadal axis, the improvement of the germinative epithelium was better than in boys with suspected gonadotropin deficiency (69). Hypogonadism may also to some extent be sequelae from the cryptorchidism abnormality itself (70). Several studies have suggested that cryptorchidism, particularly bilateral cryptorchidism, is associated with reduced spermatogenesis and inhibin-B levels and increased FSH levels in adulthood (4, 71–73). Similar hormonal findings have been observed during mini-puberty and puberty in some studies (74, 75). Several studies have suggested reduced Leydig cell function during mini-puberty, at least in more severe forms of cryptorchidism (76–78). Such cases may not only have reduced germ cell number at birth, but also have congenital impairment of Leydig cell function. It has also been shown that the Sertoli cell number is reduced in some cryptorchid testes from boys less than 1 year old compared to normal (79). So, the whole testis may be congenitally affected.

**Table 1:** The hormonal parameters, germ cell numbers per tubular transverse sections and Ad Spermatogonia numbers per tubular transverse sections from 10 boys with bilateral cryptorchidism and suspected gonadotropin insufficiency at primary orchidopexy and testicular biopsy for cryopreservation median 15 months after primary surgery (68).

patient No	age at orchidopexy months	age at second biopsy months	FSH-1 IU/L	FSH-2 IU/L	LH-1 IU/L	LH-2 IU/L	inhibin B-1 pg/mL	inhibin B-2 pg/mL	mean G/T 1	mean G/T 2	mean AdS/T 1	mean AdS/T 2
1	26	45	0.6	0.7	0.1	0.7	80	61	0.24	0.41	0.050	0.019
2	12	27	0.6	1.2	0.1	0.3	165	–	0.06	0.19	0.000	0.000
3	10	24	1.4	1.7	0.1	0.1	126	132	0.07	0.09	0.000	0.006
4	45	63	0.6	0.3	0.1	0.1	17	28	0.35	0.43	0.004	0.020
5	25	43	0.7	2.1	0.1	1.4	118	74	0.06	0.67	0.004	0.051
6	6	16	0.8	0.5	0.1	0.4	300	48	0.07	0.17	0.000	0.000
7	13	32	0.9	0.8	0.1	0.1	44	32	0.03	0.03	0.000	0.000
8	20	32	1.0	0.7	0.1	0.1	69	38	0.05	0.22	0.000	0.013
9	34	41	0.6	1.3	0.1	0.3	69	111	0.34	0.31	0.018	0.013

However, there are also a few studies showing that early placement of the testes in scrotum in childhood improves fertility parameters in adult age, which indirectly points towards a better germ cell survival when heat exposure is shorter (8, 80, 81). Testicular growth shown after early surgery is primarily positive related to the testicular germ cell number. Such findings are supported by the studies from Kollin and co-workers (82, 83).

## The fate of germ cells in cryptorchid testes in respect of malignancy

**Figure 4:**
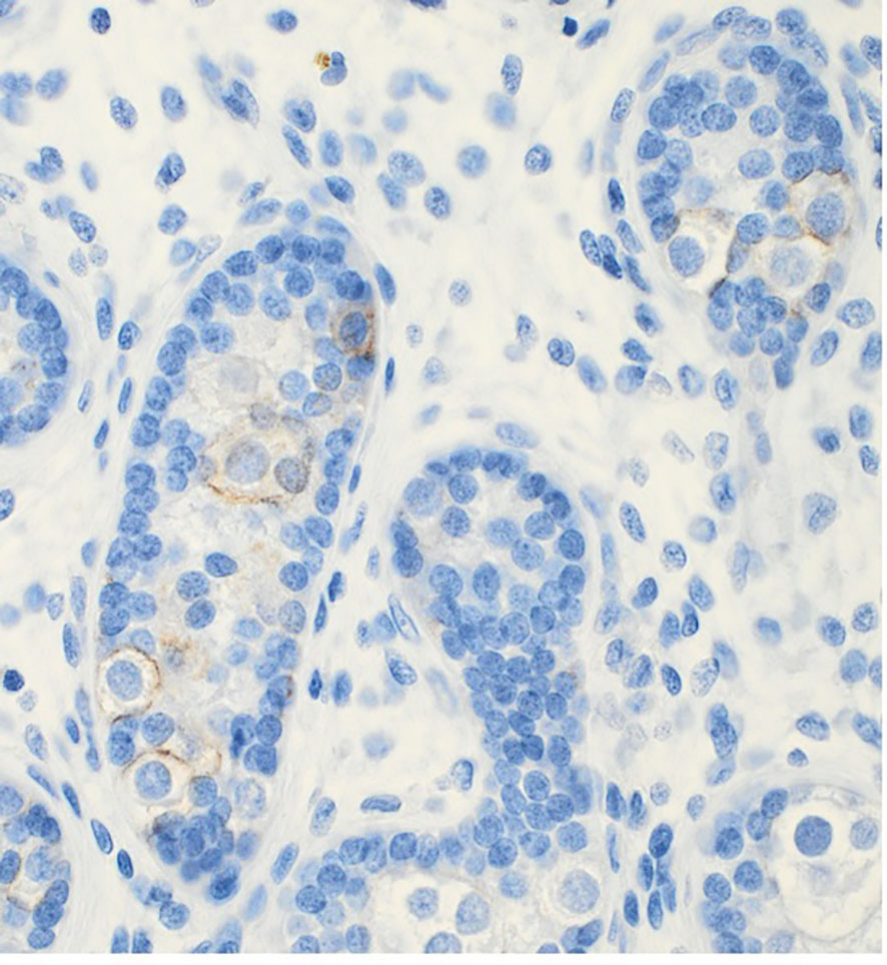
PLAP positive stained germ cells in the periphery of the tubules of a testicular biopsy from a 5 years old boy with cryptorchidism.

A hypothesis suggests that the elevated temperature experienced by cryptorchid testes can lead to aberrant apoptosis. This may allow some gonocytes to persist and evolve into GCNIS through progressive mutation and/or cellular unbalance, ultimately developing into malignancy in adulthood (84). These abnormal gonocytes are kept in a defined environment “suspended animation” in the germ cell line and, due to the accumulation of mutations, may undergo transformation becoming the source of the GCNIS (84, 85). This hypothesis aligns with recent findings that the immunohistochemical marker PLAP is normally detected in gonocytes, and the persistence of PLAP-positive germ cells frequently are found throughout all childhood in accordance with the delayed germ cell development seen in cryptorchid testes (61, 63) (**Figure 3**). However, later in childhood, these PLAP-positive germ cells are often identified in the periphery of the tubules (**Figure 4**). Thus, the term ‘PLAP-positive germ cells’ should be used instead of ‘persistent gonocytes’ for accuracy. These PLAP-positive germ cells are always Oct3/4 negative, so such PLAP-positive germ cells must just be considered as germ cells with some pluripotent properties (**Figure 4**). After puberty, PLAP-positive cells should have disappeared. An important additional factor may be associated with the hormonal changes occurring during puberty (85). When testicular cancer manifests in adulthood, the adult GCNIS could represent a new histological pattern before invasive germ cell cancer becomes evident. In post-pubertal and adult patients, the GCNIS may arise from PLAP-positive germ cells, which are often normally abundant through puberty, or in germ cells that transform into cells resembling pluripotent stem cells. PLAP-positive germ cells possess pluripotent stem cell properties. If they persist after puberty, there is a potential for them to evolve into cancer (86). Gonocytes are known to be Oct3/4 positive and have almost completely demethylated DNA, which facilitates the accumulation of mutations during cell replication and the development of GCNIS (85). GCNIS originating during fetal development may manifest in rare cases associated with disorders of sexual development (DSD), chromosomal abnormalities in boys, specific syndromes and teratomas that include cryptorchidism (7, 61, 63). However, in general, GCNIS is so rarely demonstrated in prepubertal cryptorchid testes, that it is not plausible that adult GCNIS generally originates during fetal development in non-syndromic cryptorchidism (7, 61, 63). Koni and co-workers (87) studied 51 men (aged 20–24 years) diagnosed with inguinal unilateral cryptorchidism found on routine examination for military recruits. None was evaluated or treated for cryptorchidism previously. All men had a normal contralateral testis and no other observed phenotypic alterations, and all underwent unilateral orchiectomy. With combined morphological evaluation and the use of specific adult cancer immunohistochemical markers OCT3/4 and CD117 staining, they diagnosed one case (2%) of GCNIS. The importance of using immunohistochemical staining in histologic evaluation was stressed. In a similar study, Ates and co-workers (88) found one incidentally diagnosed case with seminoma out of 244 unilateral cryptorchid men who were orchiectomized between 19 to 24 years of age. Since immunohistochemical staining was not used in that study, this may explain why they did not diagnose any cases with GCNIS. Soltanghoraee and co-workers (89) reviewed testicular biopsies of 1,153 infertile men. In this cohort, 190 patients had a history of unilateral or bilateral cryptorchidism in which 127 had undergone orchidopexy (the age of orchidopexy was known only in 95 patients; between 4–48 years of age). GCNIS was detected in 7 of the 1,153 examined patients. Six of these seven patients had a history of intra-abdominal cryptorchidism. The age at GCNIS diagnosis was 27 to 36 (median, 31) years old. Among the 190 patients with cryptorchidism, there was an GCNIS prevalence rate of 3.1%. PLAP immunohistochemical staining was used in this study. The latter data are in accordance with the risk and incidence ratios of testicular cancer in adulthood from 3 other cohort series of males with previous prepubertal surgical treatment of cryptorchidism published in a recent meta-analysis (90). In another cohort study including 1403 men operated prepubertally/pubertally for cryptorchidism with testicular biopsies taken from the cryptorchid testes the standardized incidence ratio for development of testicular cancer in adult age was 2.7 (95% CI: 1.5–4.3) (19). The lifetime risk of developing testicular cancer in the male background population does generally not exceed 1%, but may vary between regions (91).

As previously mentioned, there is good evidence that the presence of abnormal gonocytes causes neoplasia in humans with syndromic cryptorchidism, DSD and teratoma cases. Such two such cases were found in the series of Osterballe and co-workers (19). However, besides those two cases, only 1 of 13 boys, who developed testicular cancer at 25 to 37 years old had any positive staining of the original biopsy. All these patients had their prepubertal biopsy of the cryptorchid testes stained with immunohistochemical GCNIS markers. The 14-year-old patient who developed seminoma at 36 years old had originally a weak PLAP-positive staining of germ cells at the basement membrane, which is often seen in prepubertal biopsies of boys with non-syndromic cryptorchid testes. These findings can explain why the relative risk of developing testicular cancer increases when the orchidopexy was performed after puberty. So, the long-term thermal injury on any germ cells during childhood causing progressive germ cell loss/apoptosis (92), and the hormonal changes occurring during puberty may be important additional factors to be associated with the occurrence of testicular cancer later in adult cases. In adults, the presence of GCNIS predominantly represents a new histological pattern before invasive germ cell cancer is demonstrated and is neither congenital nor related to abnormal early infant gonocyte transformation. The role of persistent PLAP and c-Kit positive germ cells in normal prepubertal testes still needs to be elucidated. However, the well-documented fact that early orchidopexy decreases the risk of later testicular cancer development in adult age supports the hypothesis that when testicular cancer occurs later in adult previously cryptorchid cases, this adult GCNIS most often has a new different histological pattern (20, 21, 93). These findings concerning GCNIS in adult men are contradicting the prevailing hypothesis of fetal origin of also adult GCNIS (94, 95).

## Conclusion

Impaired germ cell number in cryptorchid testes may be congenital as it is seen already antenatally in the third trimester and in newborns. In cryptorchidism, there is a fast decline in germ cell number during the first year, that to some extent is physiologic due to the apoptosis of the gonocytes, but the germ cells do not differentiate and proliferate normally during mini-puberty, and there is also a delay in the process of clearing the gonocytes from the seminiferous epithelium. Sertoli cell only in testicular biopsies starts at 10 months of age and germ cell deterioration is progressive as about 50% of persisting cryptorchid testes in puberty have Sertoli cell only. Besides congenital abnormal cryptorchid testes, the elevated temperature of cryptorchid testes triggers cell death, impairs germ cell maturation and proliferation and has been proposed as being the cause of infertility. Moreover, hormonal deficiency also plays a role in the abnormal germ cell development. GCNIS originating during foetal development may be true in rare cases of DSD, boys with chromosomal abnormalities, specific syndromes and teratomas that includes cryptorchidism. In adults, the presence of GCNIS predominantly represents a new histological pattern before invasive germ cell cancer is demonstrated and is neither congenital nor related to abnormal early infant gonocyte transformation.

## Author contributions

JT: Writing – original draft, Writing – review & editing. SH: Writing – review & editing. AH: Writing – review & editing. JB: Writing – review & editing. LM: Writing – review & editing. CA: Writing – review & editing. TO: Writing – review & editing. DC: Writing – review & editing.
